# Searching the Internet for psychiatric disorders among Arab and Jewish Israelis: insights from a comprehensive infodemiological survey

**DOI:** 10.7717/peerj.4507

**Published:** 2018-03-14

**Authors:** Mohammad Adawi, Howard Amital, Mahmud Mahamid, Daniela Amital, Bishara Bisharat, Naim Mahroum, Kassem Sharif, Adi Guy, Amin Adawi, Hussein Mahagna, Arsalan Abu Much, Samaa Watad, Nicola Luigi Bragazzi, Abdulla Watad

**Affiliations:** 1 Padeh and Ziv Medical Centers, Azrieli Faculty of Medicine, Bar-Ilan University, Zefat, Israel; 2 Zabludowicz Center for Autoimmune Diseases, Department of Medicine B, Sheba Medical Center, and Sackler Faculty of Medicine, Tel Aviv University, Ramat Gan, Israel; 3 EMMS Nazareth Hospital, Nazareth, Azrieli Faculty of Medicine, Bar-Ilan University, Safed, Israel; 4 The Society for Health Promotion of the Arab Community, The Max Stern Yezreel Valley College, Nazareth, Israel; 5 Sackler Faculty of Medicine, Tel Aviv University, Ness Ziona-Beer Yaacov Mental Health Center, Beer-Yaacov, Tel Aviv, Israel; 6 Department of Medicine B, Sheba Medical Center, and Sackler Faculty of Medicine, Tel Aviv University, Ramat Gan, Israel; 7 Department of Statistics and Operations Research, Tel Aviiv University, Tel Aviv, Israel; 8 Department of Health Sciences (DISSAL), School of Public Health, University of Genoa, Genoa, Italy

**Keywords:** Digital divide and inequalities, Psychiatric disorders and mental health, Web searches

## Abstract

Israel represents a complex and pluralistic society comprising two major ethno-national groups, Israeli Jews and Israeli Arabs, which differ in terms of religious and cultural values as well as social constructs. According to the so-called “diversification hypothesis”, within the framework of e-health and in the era of new information and communication technologies, seeking online health information could be a channel to increase health literacy, especially among disadvantaged groups. However, little is known concerning digital seeking behavior and, in particular, digital mental health literacy. This study was conducted in order to fill in this gap. Concerning raw figures, unadjusted for confounding variables (time, population size, Internet penetration index, disease rate), “depression” searched in Hebrew was characterized by 1.5 times higher search volumes, slightly declining throughout time, whereas relative search volumes (RSVs) related to “depression” searched in Arabic tended to increase over the years. Similar patterns could be detected for “phobia” (in Hebrew 1.4-fold higher than in Arabic) and for “anxiety” (with the searches performed in Hebrew 2.3 times higher than in Arabic). “Suicide” in Hebrew was searched 2.0-fold more than in Arabic (interestingly for both languages search volumes exhibited seasonal cyclic patterns). Eating disorders were searched more in Hebrew: 8.0-times more for “bulimia”, whilst “anorexia” was searched in Hebrew only. When adjusting for confounding variables, association between digital seeking behavior and ethnicity remained statistically significant (*p*-value < 0.0001) for all psychiatric disorders considered in the current investigation, except for “bulimia” (*p* = 0.989). More in details, Israeli Arabs searched for mental health disorders less than Jews, apart from “depression”. Arab and Jewish Israelis, besides differing in terms of language, religion, social and cultural values, have different patterns of usage of healthcare services and provisions, as well as e-healthcare services concerning mental health. Policy- and decision-makers should be aware of this and make their best efforts to promote digital health literacy among the Arab population in Israel.

## Introduction

Israel represents a complex, multicultural and pluralistic society where sometimes clashing and opposite tendencies coexist. More in detail, Israel comprises two major ethno-national groups, Israeli Jews and Israeli Arabs, with about 79% of the population being Jewish. The two groups differ in terms of religious and cultural values as well as social constructs: for instance, Israeli Arabs hold a traditional collectivist values, highly cohesive culture, whereas Israeli Jews are more imbued with Western codes and influences. Furthermore, the two groups reside in geographically different areas and settings, and utilize separate social and cultural networks, such as schools, educational and religious institutions, as well as mass media and other channels (Abbas & Mesch, 2015; Mesch, 2016).

In addition, these differences reflect in health literacy and in the use of healthcare services: Israeli Arabs tend to underutilize healthcare facilities and support with respect to Israeli Jews. Different factors may explain this, including lack of proper information and knowledge concerning the delivery of health provision, perceived barriers, like language and stigma, as well as a preference towards non-conventional treatments (such as religious management of the disease) and informal support (Al-Krenawi, 2002; Ayalon et al., 2015; Baron-Epel, Garty & Green, 2007; Clarfield et al., 2017; Khatib, Roe & Yerushalmi, 2016; Southern et al., 2015). Specifically concerning mental health services, Arab-Israeli patients tend to contact physicians with a two-fold delay compared to Jews, due to lower schooling and distrust in the treatment (Ponizovsky et al., 2007).

A divide between Israeli Arabs and Israeli Jews exists as well in the use of the Internet. Mesch & Talmud (2011) recruited a representative sample of 1,374 Israelis and found that Israeli Jews reported to use information and communication technologies and to access the Internet more than Israeli Arabs (approximately 72% *versus* 53%, respectively, statistically significant with a *p*-value < 0.001). This digital divide was found to be complex and multi-factorial, depending on compositional effects (human capital, education and income), categorical effects (occupational structure), and motivational factors (attitudes towards technologies). Interestingly, also the pattern of digital seeking information is different: Arabs tend to use more collective sites, such as blogs, forums and social media/networks, with respect to Jews (Avidar, 2009).

Despite such quantitative and qualitative differences, according to the so-called “social diversification hypothesis”, “minorities and immigrants will be more likely to use computer-mediated communication to compensate for their lack of social capital” (Mesch, Mano & Tsamir, 2012), within the framework of e-health and in the era of new information and communication technologies. Online health information seeking could be, indeed, a channel to increase health literacy and healthcare empowerment, by expanding the contacts and interactions between patients and healthcare providers, especially among disadvantaged groups (Mesch, Mano & Tsamir, 2012). Different variables, including age, gender, socio-economic status and educational level, may impact on digital behaviors and online activities. Mesch, Mano & Tsamir (2012) in a sample of 1,371 Israelis found that less advantaged groups tend to seek more online health information, even if, contrary to what expected, they use less e-healthcare services with respect to other groups.

Besides Israel, seeking information behavior has been investigated in other multicultural contexts (Fairlie, 2007), such as the United States. For example, Peña-Purcell (2008) found that Hispanics, while agreeing that the Internet represents a valuable resource for health information, accessed online health information less than non-Hispanic whites (28.9% *versus* 35.6%). Similarly, Livingston, Minushkin & Cohn (2008) found that 35% of Hispanics *versus* 71% of non-Hispanic whites searched the Internet for health information. A more subtle finding was obtained by Lorence, Park & Fox (2006), who observed a complex interplay between ethnic and income differences in terms of access to online health information.

Specifically concerning mental health issues, Neumark et al. (2013) performed a survey among a nationally representative sample of 7,028 Israeli Jews and Arabs 7th-through 12th-grade students from 158 schools and assessed online health information seeking behavior in terms of patterns and determinants. Authors found that Arab students (63%) were more likely than Jewish students (48%) to surf the Internet for general health-related information, as well as for mental health-related information (31% *versus* 19%, *p*-value < 0.0001 and 34% *versus* 23%, *p*-value < 0.001, for Arab and Jewish boy and girl students, respectively).

However, whilst patterns of utilization of healthcare provision by Arab and Jewish residents in Israel have fostered a huge body of research, little is known concerning digital seeking behavior and, in particular, digital mental health literacy. This study was conducted in order to fill in this gap of knowledge. Thus, the main purpose of this study was to investigate the pattern of accessing the Internet for mental health-related information among Israeli Arabs and Jews, namely, how ethnicity impacts on mental health information seeking behavior.

## Material and Methods

GT is a freely available tool (accessible at https://trends.google.com/trends/) that enables scholars to track and monitor web searches related to a given topic. In the extant scholarly literature, GT has been exploited in order to investigate public interest mainly towards infectious diseases (Nuti et al., 2014) and, specifically concerning psychiatric disorders, suicide, non-sucidal self-injury (NSSI), schizophrenia, and substance use, among others (Bragazzi, 2013; Fond et al., 2015; Gahr et al., 2015; Gamma et al., 2016; Koburger et al., 2015; Saha et al., 2017; Solano et al., 2016; Tran et al., 2017).

In this study, GT was mined from inception (1st January 2004) until 30th December 2016 on 19th February 2018, searching for the most common neuropsychiatric disorders, both in Arabic and Hebrew. Searches were limited to Israel. Mental disorders were back-translated from English by native Arabic and Hebrew speakers. Different alternative translations (various possible spelling names and synonyms) have been utilized and validated by an expert Israeli psychiatrist (DA), who works with both Arab and Jewish patients.

Web queries are reported by GT not as absolute, but as normalized figures (termed as relative search volumes or RSVs). In detail, every performed query is divided by the total searches performed in that given country and time window, and then, scaled on a range from 0 to 100.

Searches can be performed using two different strategies, namely the “search term” and the “search topic” options. Whilst the first approach enables to search exactly the keyword or keywords entered by the user, the second search strategy results into a broader search, in which GT does not limit to the entered keyword(s) but systematically performs a search of all web searches containing related pertinent terms. For the current analyses, “search term” option was utilized (Bragazzi et al., 2016).

Before commencing any statistical analysis, GT-generated data were visually inspected for outliers. Log-linear robust Poisson regression analyses were carried out adjusting for confounding variables such as time (year), population size, Internet penetration index (data taken from the Israel Central Bureau of Statistics) and disease rate (data taken from relevant nation-wide studies or from the Health Ministry of Israel). These data are provided as raw data in the Supplemental Material. Poisson regression analysis has been chosen in light of the particular kind of data provided by GT itself; normalized and scaled figures of website traffic and search engine volumes, amassed from users over a certain time period and spatial location, and aggregated on a given time basis (in the current cause, on a yearly basis).

Figures with *p*-value less than 0.05 were considered statistically significant. XLSTAT software (XLSTAT Premium version 19.7 for Windows, Addinsoft, France) was utilized.

## Results

**Figure 1:**
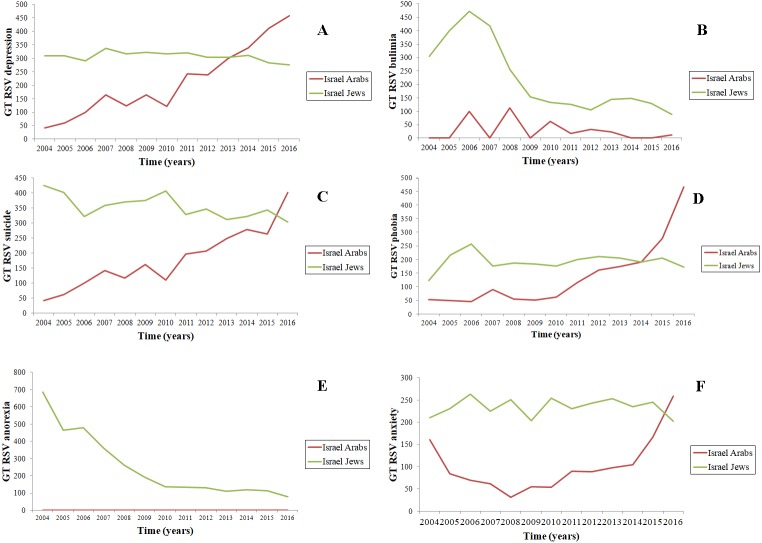
Plots showing the trends of web searches related to psychiatric disorders (namely, (A) depression, (B) bulimia, (C) suicide, (D) phobia, (E) anorexia and (F) anxiety) as captured by Google Trends (GT).

Concerning raw figures, unadjusted for confounding variables (time, population size, Internet penetration index, disease rate), “depression” searched in Hebrew was characterized by 1.5 times higher search volumes, slightly declining throughout time, whereas RSVs related to “depression” searched in Arabic tended to increase over the years. Similar patterns could be detected for “phobia” (in Hebrew 1.4-fold higher than in Arabic) and for “anxiety” (with the searches performed in Hebrew 2.3 times higher than in Arabic). “Suicide” in Hebrew was searched 2.0-fold more than in Arabic (interestingly for both languages, search volumes exhibited seasonal cyclic patterns). Eating disorders were searched more in Hebrew: 8.0-times more for “bulimia”, whilst “anorexia” was searched in Hebrew only (Table 1, Fig. 1).

When adjusting for confounding variables, association between digital seeking behavior and ethnicity remained statistically significant (*p*-value < 0.0001) for all psychiatric disorders considered in the current investigation, except for “bulimia” (*p* = 0.989; Table 2). Therefore, Israeli Arabs searched for mental health disorders less than Jews, apart from “depression”. For further details, the reader is referred to Table 2.

**Table 1:** Most common psychiatric disorders searched in Arabic and Hebrew languages, during the study period (2004–2016) as captured by Google Trends (GT).

**Psychiatric disorder**	**Time trend**	**RSV**
**Arabic language**		
Depression	Increasing	212.56 ± 132.77; 164,67 [40,83–457.50]
Suicide	Increasing	179.27 ± 100.75; 162.50 [41.50–401.50]
Anxiety	Increasing	101.62 ± 61.45; 89.00 [31.00–259.25]
Anorexia	–	–
Phobia	Increasing	137.51 ± 122.27; 89.71 [45.14–467.00]
Bulimia	Stable	27.69 ± 39.56; 11.00 [0.00–113.00]
**Hebrew language**		
Depression	Stable	307.60 ± 16.46; 309.67 [276.56–336.44]
Suicide	Stable	355.38 ± 38.68; 346.67 [304.33–425.00]
Anxiety	Stable	234.54 ± 19.88; 235.25 [202.25–263.25]
Anorexia	Decreasing	251.00 ± 189.27; 136.00 [78.00–686.00]
Phobia	Stable	192.51 ± 30.74; 191.17 [121.83–256.17]
Bulimia	Decreasing	221.54 ± 133.80; 148.00 [88.00–473.00]

**Table 2:** Log-linear Poisson robust regression analyses showing the association between ethnicity and web searches related to psychiatric disorders, correcting for time (year), population size, Internet penetration index and disease rate as confounding factors.

**Source**	**Value**	**Standard error**	**Wald Chi-Square**	**Pr > Chi^2^**	**Wald lower bound (95%)**	**Wald upper bound (95%)**
**Depression**						
Arab Israelis *vs* Jewish Israelis	0.778	0.153	25.680	**<0.0001**	0.477	1.078
**Anxiety**						
Arab Israelis *vs* Jewish Israelis	−5.331	0.295	326.390	**<0.0001**	−5.910	−4.753
**Phobia**						
Arab Israelis *vs* Jewish Israelis	−4.173	0.255	267.151	**<0.0001**	−4.674	−3.673
**Anorexia**						
Arab Israelis *vs* Jewish Israelis	−25.124	1896.315	0.000	0.989	−3741.834	3691.586
**Bulimia**						
Arab Israelis *vs* Jewish Israelis	−3.134	0.244	165.195	**<0.0001**	−3.612	−2.656
**Suicide**						
Arab Israelis *vs* Jewish Israelis	−4.396	0.193	518.492	**<0.0001**	−4.774	−4.017

## Discussion

The present study systematically investigated differences in digital seeking behavior related to common psychiatric disorders among Arab and Jewish Israelis characterized by different disease rates: for two of these diseases (namely, phobia and anxiety) rate was comparable among the two populations. Iancu et al. (2011) recruited a sample of 153 Jewish and 147 Arab students and found that social anxiety disorder did not differ between the two groups. According to a community-based study performed by Levav et al. (2007), twelve-month prevalence rates for anxiety were not significantly higher among Arab Israelis, even though rates of help-seeking from specialized health services were found to be lower among Arab Israelis. Disease rate was, instead, higher among Jews for anorexia, bulimia and for suicide. Concerning eating and weight disorders, several studies found ethnic differences in attitude toward food (Apter et al., 1994; Goldzak-Kunik & Leshem, 2017; Kaluski et al., 2008; Latzer, Witztum & Stein, 2008), with Jewish girls exhibiting anorectic- or bulimic-like eating patterns. Similarly, suicide and suicidal ideation tended to occur less frequently in the Arab Israeli population (Brunstein Klomek et al., 2016; Gofin et al., 2000; Gvion, Levi-Belz & Apter, 2014; Lubin et al., 2001; Morad et al., 2005). In the case of depression, disease rate was higher among Arabs: Kaplan et al. (2010) found that the rate of depression scores was 2.5 times higher among Arabs than among Jews, with women being more likely to express symptoms of depressive episode than men and with depression scores increasing throughout the years.

Psychiatric disorders are complex and multifactorial diseases that arise from the non linear interplay between culture, religion, personality, biological make-up and environmental situations (for example, in Israel, geopolitical events) (Baker & Shalhoub-Kevorkian, 1999).

The different social and cultural characteristics of lifestyle and the social control systems among Jewish and Arab Israelis may explain the attitudes towards mental health (Ashkar et al., 2006; Katz-Sheiban & Eshet, 2008; Levav & Aisenberg, 1989) as well as towards eHealth (Neter & Brainin, 2012).

Ethnic differences can exist for some psychiatric disorders and for mental health literacy and help seeking/online health information seeking. According to the “diversification hypothesis”, Israeli Arabs would be expected to exploit new information and communication technologies (ICTs) in order to gather more insights concerning psychiatric diseases more than Jews. However, our findings showed that web searches related to mental disorders with the exception of “depression” and of “anorexia” were performed much more in Hebrew rather than in Arabic in a statistically significant way, emphasizing the need to promote digital mental health literacy among Arab Israelis. On the other hand, it is intriguing to notice that web searches exhibited a decreasing time trend when searched in Hebrew, with respect to the web queries carried out in Arabic.

Our study presents some strengths, like the comprehensive, systematic search of all the most common psychiatric disorders, and the novelty of the investigation. To the best of our knowledge, it is the first study to utilize GT in order to investigate online health information behavior in terms of ethnic groups. On the other hand, it has some shortcomings, which should be properly acknowledged. The major limitation is given by the fact that Arabs in Israel might search also in Hebrew, thus increasing the percentage of search volumes in Hebrew. On the other hand, only 17.4% of Israeli Arabs consume material written in Hebrew (Avidar, 2009). Another drawback is that GT provides scholars with relative, normalized values and not with raw, absolute figures, which could be further statistically handled, processed and manipulated.

## Conclusions

Arab and Jewish Israelis, besides differing in terms of language, religion, social and cultural values, have different patterns of usage of healthcare services and provisions, both online and offline. The digital divide between Israeli Jews and Arabs represents a major social inequality. Health authorities and decision-makers should be aware of this and make their best efforts to identify factors underlying online health information seeking behavior in order to promote digital mental health literacy among the Arab population in Israel and therefore to improve their health status and reduce health disparities.

##  Supplemental Information

10.7717/peerj.4507/supp-1 Data S1 Raw data: Google Trends generated data for mental health disorders, population size, time (year), Internet penetration index and disease rate Google Trends generated data for mental health disorders, population size, time (year), Internet penetration index and disease rate, showing the impact of ethnicity on online mental health seeking behavior.

## Additional Information and Declarations

### Data Availability

The following information was supplied regarding data availability: The raw data are included as Supplemental File.
